# Bite of a Rattlesnake

**Published:** 1853-04

**Authors:** Thomas A. Atchison


From the Southern Journal.

*Bite of the Rattlesnake.*

Messrs. Editors: I believe as yet no distinguished authority has elevated the claims of Alcohol to the character of specific in this terrible malady; a character to which I think it justly entitled, as the following case will show:

I was summoned in haste on the evening of the 20th September, 1852, to see Miss R——, a young lady aged 17, living five miles in the country, (I was informed by the messenger,) while taking a stroll in company with her mother, was bitten by a Rattlesnake. I arrived at half past seven o'clock, two hours and a half after the accident. I found my patient almost moribund, pulse wavy and scarcely perceptible at the wrist, surface cold and bathed in perspiration, face swollen with a besotted expression, mind wavering, pupils dilated, could not see, declaring that it was very dark, although candles were burning in the room, asked frequently if it was not raining hard, although the night was calm and clear.—Upon examination I found that the bite had been inflicted on the instep of the left foot; two little punctures were very perceptible, around which there was a greenish areola, with some puffiness.

Having heard of the marvelous efficacy of "spirits" in the relief of similar cases, I at once determined to give the remedy a full and fair trial. Reason and analogy sustained it. The nervous system was overwhelmed by a swift and deadly sedative poison, it must be supported by an equally powerful *diffusible* stimulant; accordingly I gave half a glass of whisky, which was swallowed with avidity. Meanwhile the wound was freely scarified and cupped, and the extremities placed in a hot saline bath; twenty grains of carb. ammonia was then given, which was immediately thrown up, together with the contents of the stomach, colored a bright grass green. A common sized glass full of whiskey was now given, the patient draining with eagerness the last drops, and begging with the energy of instinct for more; thus a glass of whisky and twenty grs. of carb. ammonia were given alternately every half hour, until three pints of the former and eighty grs. of the latter were taken; and what is remarkable, not the slightest intoxication ensued, on the contrary, the urgent and alarming symptoms gradually gave way, warmth was restored to the surface, the pulse returned to the wrist, the mind was called back from its wanderings, and she fell into a quiet sleep, from which she awoke at five o'clock, A. M., complaining of immense pain in the foot, shooting up the inside of the leg to the knee: ordered, morphia one-fourth gr., fomentation of laudanum and camphor, followed by poultice of linum lini, witht he effect of entire relief of pain.—The following day castor oil was given to move the bowels; from that hour she suffered no further inconvenience from the bite.

The instinctive avidity and impunity with which this delicately nurtured young lady took so large a quantity of spirits, sufficient under ordinary circumstances to have killed a regular *habituc*, would excite astonishment, if we did not reflect that it was antagonized by the depressing effect of the poison on the nervous system.

But the most interesting feature in this case remains to be stated. Miss R——, at the time she was bitten, was the subject of well marked whooping-cough, which was then epidemic in the neighborhood. She had had the disease about three weeks, consequently it was at its acme, but on recovering from the effects of the poison, to her great surprise and gratification, her cough had disappeared also, nor did it return. Being a spasmodic disease, it was swept away by the powerful impression made upon the nervous system.

This interesting and novel fact, it seems to me, furnishes some hints in the treatment of this hitherto intractable malady—hooping-cough, some remarks upon which I reserve for a future communication. Yours truly,

THOMAS A. ATCHISON.

